# Overcoming cathode coating inhomogeneity: the role of LiF interlayer in enabling conformal LiNbO_3_ protection on LiNi_0.8_Co_0.1_Mn_0.1_O_2_ for all solid-state batteries

**DOI:** 10.1039/d6ta03220f

**Published:** 2026-07-24

**Authors:** Hari Vignesh Ramasamy, Robin N. Wullich, Barthélémy Lelotte, Valerie Siller, Carlos A. F. Vaz, Elisabeth Müller, Mario El Kazzi

**Affiliations:** a PSI Center for Energy and Environmental Sciences, Paul Scherrer Institute 5232 Villigen PSI Switzerland mario.el-kazzi@gmail.com; b Swill Light Source, Paul Scherrer Institut 5232 Villigen PSI Switzerland; c PSI Center for Life Sciences, Paul Scherrer Institute 5232 Villigen Switzerland

## Abstract

Ni-rich cathode active materials (CAMs) paired with argyrodite solid electrolytes (SEs) such as Li_6_PS_5_Cl (LPSCl) are promising for high-energy density and inherently safer all-solid-state batteries (ASSBs). However, severe (electro-)chemical incompatibilities at the CAM/SE interface lead to parasitic reactions and the formation of resistive interphases. These interfacial degradations impede lithium-ion transport, limit rate capability, and ultimately trigger capacity fading upon cycling. Therefore, developing a stable, ionically conductive, and chemically robust CAM/SE interface remains a critical challenge. In this work, we report an efficient dual-surface-coating strategy based on ultrathin LiF and LiNbO_3_ layers applied to LiNi_0.8_Co_0.1_Mn_0.1_O_2_ (NCM811) cathode particles. Surface fluorination *via* a controlled CHF_3_ gas-phase reaction converts adventitious Li_2_CO_3_ into a conformal LiF inner layer, while simultaneously promoting the formation of a uniform LiNbO_3_ outer coating. This synergistic LiF/LiNbO_3_ architecture effectively protects the CAM surface while facilitating fast interfacial lithium-ion transport. As a result, the modified NCM811 cathode exhibits markedly reduced interfacial resistance and significantly improved specific capacity at high current densities when coupled with LPSCl. Comprehensive structural, chemical, and electrochemical characterization provided in this study elucidates the pivotal role of a homogenous coating in stabilizing the CAM/SE interface, offering a viable pathway toward durable, high-performance Ni-rich ASSBs.

## Abstract 2

A dual LiF/LiNbO_3_ coating on NCM811 converts resistive Li_2_CO_3_ into a protecting fast ion conducting shield. When shield with Li_6_PS_5_Cl solid electrolytes, it suppresses interfacial degradation and boosts rate capability for durable ASSBs.

## Graphical abstract

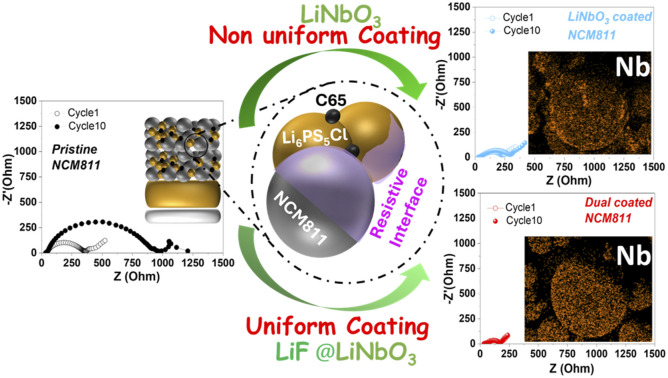

The transition toward climate neutrality requires a substantial reduction in the reliance on fossil fuels and a rapid expansion of renewable energy technologies.^1^ At the same time, the electrification of transportation, the rapid growth of portable electronic devices, and the emergence of mobile robotics and autonomous systems are driving an unprecedented demand for advanced energy storage. These applications require batteries that combine high energy density, long cycle life, fast charging capability, and uncompromising safety, while maintaining compact form factors and reliable operation across diverse conditions. However, conventional lithium-ion batteries employing flammable liquid electrolytes are approaching their theoretical energy-density limits and continue to face critical safety concerns. These limitations have driven intensive research efforts toward all-solid-state batteries (ASSBs), which are widely regarded as promising next-generation energy storage systems due to their potential for enhanced safety, higher energy density, facile bipolar cell stacking, and the utilization of high-capacity lithium metal anodes.^2,3^

Inorganic solid-state electrolytes (SEs) can be broadly classified into oxide, sulfide, and halide based materials.^4^ Among them, sulfide-based electrolytes are particularly attractive, as they exhibit exceptionally high ionic conductivity at room temperature (10^−3^–10^−2^ S cm^−1^) and possess relatively low elastic moduli, which enable cold pressing and improved interfacial contact with the cathode active materials (CAMs).^5,6^ Despite these advantages, sulfide SEs suffer from a narrow electrochemical stability window, typically below ∼2.8 V *vs.* Li^+^/Li. At higher potentials, oxidative decomposition occurs, predominantly during the initial charge, leading to the formation of ionically resistive bridging sulfur-rich interphase species.^7^ Beyond electrochemical oxidation, parasitic chemical reactions between the sulfide SEs and the surface of the CAMs further compromise interfacial stability. These reactions induce the formation of an electrochemically inactive surface, caused by the formation of reduced transition metals and oxygen loss from the layered oxide cathodes, resulting in sulfide/sulfate- and phosphide/phosphate-based decomposition products.^8,9^ The continuous growth of such interfacial reaction layers progressively increases interfacial resistance, impedes lithium-ion transport, degrades rate capability, and ultimately leads to pronounced capacity fading during cycling.

To address interfacial instability in sulfide-based ASSBs, surface coating of CAMs has emerged as the most widely adopted mitigation strategy. In this approach, a thin and conformal buffer layer is introduced between the CAM and the sulfide electrolyte. Ideally, such CAMs coatings exhibit high lithium-ion conductivity, negligible electronic conductivity, and strong (electro-)chemical compatibility with both the CAM and the SE. When effective, surface coatings suppress parasitic interfacial reactions, reduce interfacial charge-transfer resistance and its evolution upon cycling, enhance high-rate performance, and improve long-term cyclability.^10^ To date, a variety of inorganic coating materials, including Li_2_ZrO_3_,^11^ Li_2_SiO_3_,^12^ Li_4_Ti_5_O_12_,^13^ LiNbO_3_,^14^ LiTaO_3_,^14^ and related compounds, have been reported to significantly improve interfacial stability and electrochemical performance in sulfide-based ASSBs.

However, the practical effectiveness of these coatings is often limited by the deposition techniques employed. Conventional wet chemical methods,^15^ spray coating or atomic layer deposition^16^ techniques frequently involve complex processing routes or may lead to non-uniform, discontinuous, or inhomogeneously thick coatings. Such imperfections compromise the protective function of the coating layer and limit the achievable electrochemical improvements. Furthermore, for high-nickel layered oxide cathodes, unavoidable air exposure during processing leads to the formation of adventitious Li_2_CO_3_ surface impurities, which hinder lithium-ion transport and are particularly detrimental to ASSB performance.^17^

These carbonates are electrochemically oxidized during cycling, triggering gas evolution (O_2_ and CO_2_) and a cascade of other parasitic reactions involving the electrolyte in combination with other cell components.^18^ Hence, mitigating this Li_2_CO_3_ impurity and improving the homogeneity of the surface coating represent a critical pathway forward.

In this study, LiNbO_3_ is selected as a representative coating material and a model system for the broader class of oxide-based interfacial coatings. LiNbO_3_ is widely considered an effective surface coating owing to its favorable balance of ionic and electronic transport properties, particularly in its amorphous state.^19–21^ Nevertheless, most reported LiNbO_3_ coatings prepared *via* conventional methods suffer from poor uniformity and incomplete surface coverage, underscoring the need for improved coating strategies to fully exploit its interfacial stabilization potential.^22–24^

Fig. 1 illustrates the NCM811 coating workflow and simplified synthesis steps used to realize both the single-layer LiNbO_3_ coating and the dual-layer LiF|LiNbO_3_ coating consisting of an inner LiF layer and an outer LiNbO_3_ layer. The dual-coating approach simultaneously addresses two key challenges: the removal of the thin adventitious Li_2_CO_3_ contaminants that ubiquitously cover the surface of the vast majority of the layered oxide cathode materials, and the improvement of LiNbO_3_ coating homogeneity typically obtained *via* conventional sol–gel methods. In the first step, the surface of NCM811 was fluorinated using a mild fluorination agent, CHF_3_ gas, which is non-toxic, non-corrosive, and non-flammable at room temperature. During this treatment, Li_2_CO_3_ was converted into a nanometer-thick LiF layer at 350 *°*C. This fluorination strategy, developed in our earlier work, represents an innovative route to repurpose highly potent greenhouse gases such as CHF_3_, commonly generated as byproducts in the manufacturing of polytetrafluoroethylene (PTFE), polyvinylidene fluoride (PVDF), and polymer foams, into value-added functional materials.^25^ As a result, a smoother cathode surface free from lithium carbonate agglomerates was obtained and covered instead by an ultra-thin LiF layer of approximately 2 nm. Following this step, an outer LiNbO_3_ coating layer (1 wt%) was deposited *via* a chemically activated sol–gel process optimized in our previous work.^15^ The chemical composition, crystal structure, and morphology of the LiF|LiNbO_3_ dual-coated samples were characterized using X-ray diffraction (XRD), scanning electron microscopy (SEM) coupled with energy dispersive X-ray (EDX) analysis, and transmission electron microscopy (TEM), and directly compared with their single-layer LiNbO_3_ counterparts. Finally, pristine, single-layer LiNbO_3_-coated, and LiF|LiNbO_3_ dual-coated NCM811 cathodes were electrochemically evaluated in Li_6_PS_5_Cl (LPSCl)-based ASSB cells using an InLi_*x*_ counter electrode. Cells were cycled up to 4.3 V *vs.* Li^+^/Li, and their performance was assessed by galvanostatic cycling, cyclic voltammetry, and electrochemical impedance spectroscopy (EIS).

**Fig. 1:**
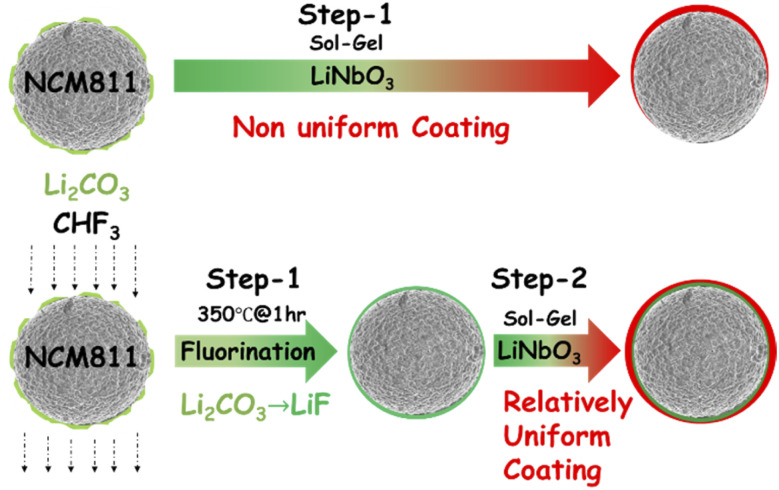
Schematic of the single-layer LiNbO_3_ coating and dual-layer LiF|LiNbO_3_ coating on CAMs.

The NCM811 cathode particles are polycrystalline, spherical in morphology, composed of primary (∼500 nm) and secondary particles (D50) of 10.0 ± 2.0 µm, and were used as received from MSE Supplies. The XRD patterns (Fig. S1) of the pristine and LiF|LiNbO_3_ dual-coated NCM811 were compared to examine any possible structural changes or the emergence of additional peaks after surface modification. All diffraction reflections peaks can be indexed to the *R*3̄*m* space group with a layered NaFeO_2_ type structure.^26^ The absence of any additional peaks confirms the amorphous nature of the coating and indicates that it does not alter the bulk crystal structure of the NCM811 cathode material. The morphology and spatial distribution of the coating were investigated using SEM coupled with EDX analysis at Nb Lα. To further substantiate the morphological observation and confirm the homogeneity of the surface coating, Gaussian Kernel density (GKD) maps of Nb L*α* were calculated at higher SEM magnifications on single NCM811 particles. A detailed description of this methodology is provided in our previous work.^15^Fig. 2(a–c) shows the surface of the 1 wt% LiNbO_3_-coated NCM811 where the niobium distribution is clearly non-uniform, with visible agglomerates dispersed across the particle surface. In contrast, Fig. 2(d–f) shows the surface of the LiF|LiNbO_3_ dual-coated NCM811, which exhibits a more uniform Nb elemental distribution without noticeable agglomeration. These observations indicate that surface fluorination plays a significant role in enhancing coating homogeneity. The microstructure of the LiF|LiNbO_3_ dual coating was further examined using TEM, which confirmed the presence of a uniform LiNbO_3_ coating layer. Fig. 2g presents the dark-field scanning TEM image (DF-STEM) of secondary NCM811 particles coated with an amorphous layer with a thickness of ∼20 nm. The corresponding elemental distribution of the constituent elements is shown in Fig. 2(h–l). Nickel, cobalt, and manganese are distributed uniformly throughout the bulk of the cathode. A distinct Nb-rich region is clearly detected and corresponds to the LiNbO_3_ coating layer. In contrast, the LiF layer, formed *via* gas-phase fluorination of CHF_3_, is estimated to be thinner than 2 nm and therefore cannot be distinctly resolved in TEM-EDX mapping due to its low thickness and limited scattering contrast. To further confirm the bilayer coating configuration, additional DF-STEM and EDX line-scan analysis were performed (Fig. S10). The elemental profiles reveal the presence of a thin LiF rich interfacial layer with a thickness of approximately 1 to 2 nm nanometers located between the NCM811 surface and the outer LiNbO_3_ coating. Above the LiF layer, a distinct Nb-enriched region with a thickness of approximately 20 nm is observed, confirming the formation of a continuous LiF/LiNbO_3_ dual coating structure. These results demonstrate that the LiF interlayer promotes the uniform growth of the LiNbO_3_ coating and contributes to the formation of a well-defined bilayer architecture. To provide direct evidence for the influence of the LiF interlayer on LiNbO_3_ coating morphology and thickness uniformity, additional characterization was performed on NCM811 particles coated solely with LiNbO_3_. Cross-sectional ion milling combined with SEM and EDX elemental mapping was employed, and the corresponding results are presented in Fig. S11. The ion-milled cross-sections enable direct visualization of the coating architecture over large particle areas while simultaneously providing elemental information through Nb mapping. As shown in Fig. S11, the single-layer LiNbO_3_ coating exhibits a highly heterogeneous morphology and thickness distribution across the NCM811 particle surface. In the absence of the LiF interlayer, the LiNbO_3_ coating displays significant local thickness variations ranging from approximately 160 to 478 nm. Furthermore, the EDX maps reveal regions containing thick Nb-rich agglomerates alongside areas with substantially thinner surface coverage, indicating incomplete coating homogeneity. These observations highlight the critical role of the LiF interlayer in regulating LiNbO_3_ deposition, suppressing the formation of large Nb-rich aggregates, and promoting the development of a thinner, more uniform, and conformal coating layer.

**Fig. 2:**
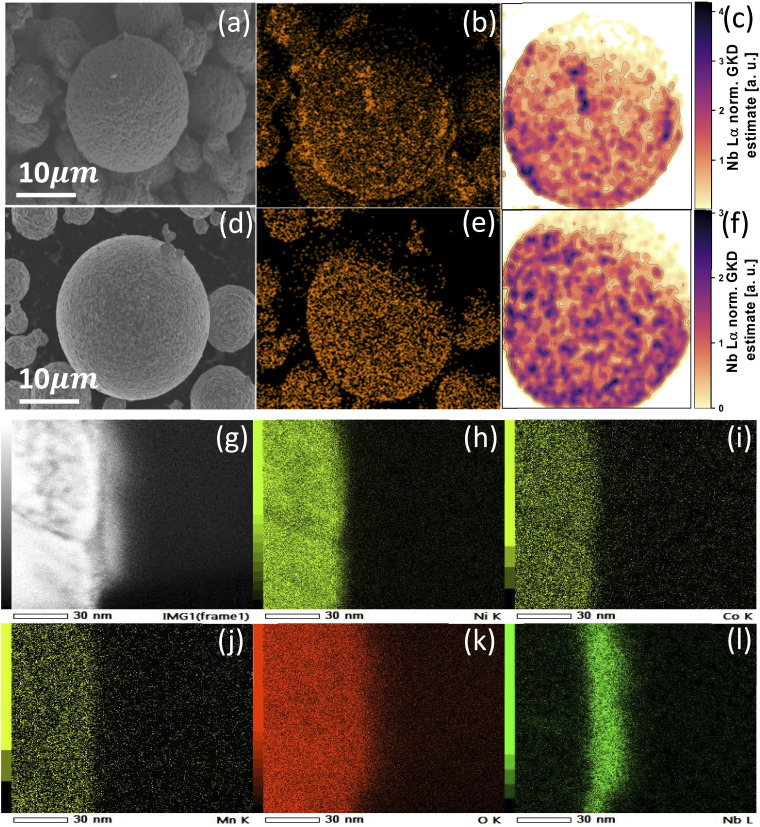
SEM image, corresponding EDX plot at the Nb Lα line, and GKD maps of the Nb Lα acquired for (a–c) single layer LiNbO_3_-coated NCM8111 and (d–f) dual layer LiF|LiNbO_3_-coated NCM811 particles. The GKD Maps were normalized to their median (*i.e.*, an estimate of one refers to the median intensity of the signal). (g) Dark-field (DF) STEM image taken on dual LiF|LiNbO_3_-coated NCM811 along with EDX mapping of the elements (h) Ni Kα, (i) Co Kα, and (j) Mn Kα (k) O Kα (l) Nb Lα.

To better understand the effect of the coating on the NCM811 surface chemistry, we performed XPS analysis on pristine, fluorinated, single-layer LiNbO_3_ and LiF|LiNbO_3_ dual-coated NCM811 particles. Fig. S2 provides the survey spectra of all four aforementioned samples. The presence of all major elements like Ni (Ni2p, Ni3p), Co (Co2p, Co3p), Mn (Mn2p, Mn3p) and O (O1s) is confirmed by their respective binding energy peaks. Fig. 3(a and b) presents the F1s spectra of the fluorinated and LiF|LiNbO_3_ dual-coated NCM811 samples. Both samples exhibit a prominent F1s peak centered at 684.3 eV, which is characteristic of LiF, confirming the successful formation of a LiF layer on the NCM811 surface during the fluorination process.^27^ In the LiF|LiNbO_3_ dual-coated sample, the persistence of the F1s signal indicates that the LiF interlayer remains intact beneath the LiNbO_3_ coating, acting as a chemically stable interfacial layer. The fitting parameters used for peak deconvolution are provided in Table S1 and Table S2.^28^

**Fig. 3:**
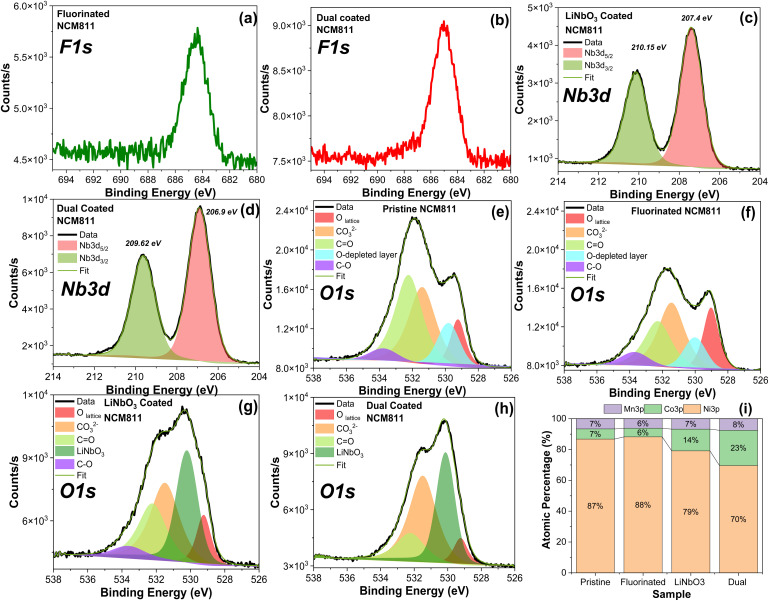
XPS analysis of pristine and surface-modified NCM811 particles. F1s core level spectra of (a) fluorinated and (b) LiF|LiNbO_3_ dual coated NCM811 particles. Nb3d core level spectra of (c) single layer LiNbO_3_ coated and (d) LiF|LiNbO_3_ dual coated NCM811. Deconvoluted O1s spectrum of (e) pristine, (f) fluorinated, (g) single layer LiNbO_3_ coated, and (h) LiF|LiNbO_3_ dual coated NCM811. (i) Atomic % of Ni3p, Co3p and Mn3p peaks estimated from the transition metal 3p region and Li1s spectra provided in Fig. S3.

The Nb3d core level spectra of single-layer LiNbO_3_-coated, and LiF|LiNbO_3_ dual-coated NCM811 were carefully fitted and are presented in Fig. 3(c and d). From the Nb3d spectra, it is evident that, the LiNbO_3_ coated NCM811 exhibits two characteristic peaks corresponding to pentavalent Nb^5+^ at 207.4 eV (Nb3d_5/2_) and 210.1 eV (Nb3d_3/2_)^15^ (Fig. 3c). With the introduction of a LiF interlayer, the Nb^5+^ peaks of the dual coated NCM811 appear at 206.9 eV and 209.6 eV (Fig. 3d). The 0.5 eV positive binding-energy shift observed for the single-layer LiNbO_3_-coated NCM811 is attributed to local variations in the morphology of the LiNbO_3_ coating rather than to a change in the Nb oxidation state. The slight binding-energy difference between the two samples is most likely associated with local charging effects arising from differences in coating thickness, uniformity, and surface coverage. Cross-sectional SEM-EDX analysis of the single-layer LiNbO_3_-coated NCM811 (Fig. S11) reveals a relatively non-uniform coating, characterized by localized LiNbO_3_ agglomerates and regions reaching several hundred nanometers in thickness. In contrast, the LiF/LiNbO_3_ dual-coated sample exhibits a significantly thinner and more homogeneous LiNbO_3_ layer (∼20 nm), as shown in Fig. 2. Given the electronically insulating nature of LiNbO_3_, variations in coating morphology and thickness can induce differential surface charging during XPS measurements. Such charging effects are expected to be particularly pronounced for photoelectrons originating directly from the LiNbO_3_ layer and can lead to apparent shifts in the measured Nb 3d binding energy.

Furthermore, the higher Nb 3d peak intensity observed for the dual-coated sample, despite the same nominal Nb loading, suggests improved surface coverage and a more uniform distribution of the LiNbO_3_ coating, with reduced agglomeration.

The O1s spectra of the pristine, fluorinated, single-layer LiNbO3-coated and LiF|LiNbO_3_ dual-coated NCM811 are shown in Fig. 3(e and h). The O1s spectrum of pristine NCM811 (Fig. 3e) consists of five major components. Lattice oxygen of NCM811 located at 529.2 eV, reduced transition metal caused by O-depleted surface at 529.7 eV.^29,30^ and three components at 531.3 eV (CO_3_^2−^), 532.2 eV (C=O) and 533.7 eV (C–O) are associated with the surface impurities such as Li_2_CO_3_/LiOH, *etc*.^15^ Upon fluorination the surface impurities are significantly reduced and a thin LiF layer forms on the cathode surface as evidenced by the reduction in the 531.4 eV (CO_3_^2−^) and 532.2 eV (C=O) peak intensities and the enhancement of the lattice oxygen signal at 530.0 eV (Fig. 3f). For the LiNbO_3_ coated sample, the oxygen depleted peak at 530.0 eV is overlapped by a new peak at a slightly higher binding energy of 530.2 eV (Fig. 3g) which corresponds to the oxygen environment in LiNbO_3_.^15^ The dual-coated NCM811 exhibits a more intense LiNbO_3_ related peak (Fig. 3h) concomitant with a significantly attenuated lattice oxygen signal. This indicates superior coating coverage and uniformity with the presence of the intermediate LiF layer as previously suggested by the Nb3d spectra. Hence the synergistic removal of resistive surface carbonates *via* fluorination and the promotion of uniform LiNbO_3_ coating growth establishes an ideal dual interface architecture.

Fig. 3(i) presents the surface composition of NCM811 cathodes derived from the area under Ni3p, Co3p, and Mn3p core level spectra (Fig. S3). The pristine material exhibits a Ni : Co : Mn atomic ratio of 87 : 7 : 7, indicating a slightly nickel-rich surface consistent with the presence of oxygen deficient spinel or rock-salt phases as inferred from the O1s peaks. After fluorination, this ratio remains almost unchanged 88 : 6 : 6. Further surface modification *via* single-layer LiNbO_3_ coating followed by post annealing at 350 °C induces a pronounced compositional change, the Ni : Co : Mn ratio changes to 79 : 14 : 7, accompanied by a substantial decrease in the surface nickel concentration and a corresponding increase in cobalt content. This trend is further amplified in the dual-coated sample where LiNbO_3_ is homogeneously covering the NCM811 surface, the ratio reaches 70 : 23 : 8. The systematic decline in Ni3p intensity, together with the increase in Co3p atomic percentage, reveals a cobalt-enrichment of the surface. Notably, the fluorination step alone (350 °C for 1 h) does not significantly alter the relative transition-metal composition detected at the surface, which suggests that the CHF_3_ treatment itself is not responsible for the observed Co enrichment. This observation indicates that the increased Co signal is more likely associated with the subsequent LiNbO_3_ coating process and the combined thermal treatments. In addition, we cannot exclude the possibility of partial Co diffusion into, or interaction with, the LiNbO_3_ coating layer during its formation and annealing. Another factor that supports this hypothesis is that after the LiF formation and removal of the native Li_2_CO_3_ layer, the LiNbO_3_ homogeneity and conformity improved. Consequently, a larger fraction of the NMC surface becomes covered by the LiNbO_3_, which may facilitate coating–cathode interactions and potentially promote enhanced local Co redistribution during the annealing process.

To better understand the effect of surface coating, the electrochemical cycling performance of pristine NCM811, single-layer LiNbO_3_ coated and LiF|LiNbO_3_ dual-coated NCM811 was investigated. For this purpose, NCM811 ASSB half cells were assembled using LPSCl as separator and InLi_*x*_ alloy as the anode. The cathode composite was prepared by mixing NCM811, LPSCl and C65 carbon in a weight ratio of 70 : 29 : 1 where C65 act as a conducting agent. The cathode active material loading was 9 mg cm^−2^. The cells were cycled at room temperature between 2.1 V and 3.7 V *vs.* InLi_*x*_ (corresponding to 2.7–4.3 V *vs.* Li^+^/Li). Fig. 4(a–d) shows the galvanostatic charge discharge voltage profiles along with long term cycling performance measured at ambient temperature for pristine NCM811, single-layer LiNbO_3_ coated NCM811 and LiF|LiNbO_3_ dual-coated NCM811 at 0.1C rate. The pristine NCM811 (Fig. 4a) delivered a first cycle charge and discharge capacity of 211.7 mA h g^−1^ and 160.5 mA h g^−1^ respectively at 0.1C rate, corresponding to an initial coulombic efficiency (CE) of 76.0%. The discharge curves of 1st, 5th and 100th cycle confirm the progressive capacity fading reaching a capacity retention of ∼80% after 100 cycles (Fig. 4d). The single layer LiNbO_3_ coated NCM811 with 1 wt% of LiNbO_3_ (Fig. 4b) exhibited an initial charge and discharge capacities of 198.4 mA h g^−1^ and 149.8 mA h g^−1^ respectively with a CE of 75.5%. Although no significant improvement in the initial CE was observed, the capacity fading after 100 cycles was mitigated, achieving a capacity retention of 89.7% (Fig. 4b).^24^ A more detailed study with 1, 2 and 4 wt% of LiNbO_3_ coating on NCM811 is provided in the supporting information (Fig. S7). The rate capability test at different current densities confirms that 1 wt% provides the optimal LiNbO_3_ coating thickness, achieving the highest capacity at high current densities and the lowest charge-transfer resistance. The LiF|LiNbO_3_ dual-coated cathode (Fig. 4c) exhibited an initial charge capacity of 210.4 mA h g^−1^ and discharge capacity of 159.5 mA h g^−1^ with the same CE of 75.8%. After 100 cycles the capacity retention was 90.0% (Fig. 4d). The coulombic efficiency evolution during the 100 cycles for the three different cathode is reported in Fig. S8

**Fig. 4:**
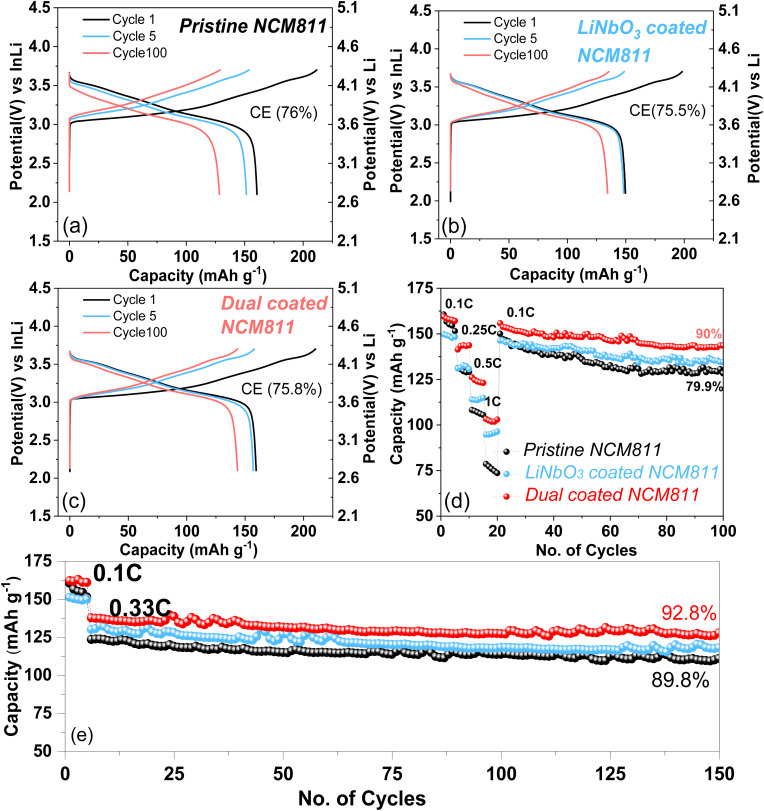
Galvanostatic charge discharge curves measured at 0.1C rate between 2.1 V to 3.7 V *vs.* InLix (2.7 V to 4.3 V *vs.* Li^+^/Li) for (a) pristine, (b) LiNbO_3_ coated and (c) dual coated NCM811. (d) Rate performance at varying current densities (from 0.1C to 1C), (e) long term cycling at 0.33C rate within 2.7 V to 4.3 V *vs.* Li^+^/Li.

Fig. 4d also compares the rate capability of the three samples evaluated at specific currents corresponding to 0.1C to 1C. The corresponding charge–discharge curves are shown in Fig. S9. While all samples exhibited decreasing capacity with increasing current rate, the coated materials significantly improved the high-rate performance. At 1C, the single-layer LiNbO_3_ coated NCM811 exhibited approximately 20% higher capacity than the pristine sample, whereas the dual-coated NCM811 showed an even greater improvement of about 31% relative to the pristine NCM811. The superior capacity of the dual-coated sample at high current densities is associated with enhanced stabilization of the CAM/SE interface in line with reduced charge transfer resistance (Fig. 5). The cyclability was further assessed at a higher current rate of 0.3C for 150 cycles (Fig. 4e) following five formation cycles at 0.1C. The initial discharge capacities of bare NCM811, single-layer LiNbO_3_ modified NCM811, and dual-coated samples were 123.5 mA h g^−1^, 130.1 mA h g^−1^, and 137.4 mA h g^−1^, respectively, with capacity retentions of 89.8%, 90.7% and 92.8% after 150 cycles.

**Fig. 5:**
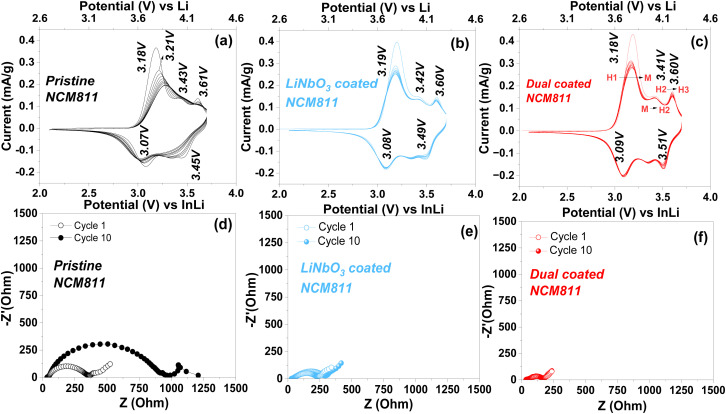
Cyclic Voltammetry curves and EIS spectrum performed on the (a and d) pristine, (b and e) single layer LiNbO_3_ coated NCM811 and (c and f) LiF|LiNbO_3_ dual coated NMC811.

Cyclic voltammetry (CV) curves of the pristine, LiNbO_3_ coated, and the dual-coated samples are shown in Fig. 5(a–c). The measurements were performed at a scan rate of 0.05 mV s^−1^ between 2.7 V and 4.3 V *vs.* Li^+^/Li. The CV curves of pristine NCM811 (Fig. 5a) exhibit three pairs of redox peaks, corresponding to transition metal redox reactions and associated structural transformations during cycling. During delithiation, oxidation peaks appear at 3.78 V, 4.03 V and 4.21 V *vs.* Li^+^/Li corresponding to Ni^2+^/Ni^3+^/Ni^4+^ and Co^3+^/Co^4+^ redox couples.^31^ They are accompanied by H1 → M, M → H2, and H2 → H3 phase transitions, where H and M represents the hexagonal and monoclinic phases, respectively.^26^ The reduction peaks appear reversibly at 3.67 V, 3.93 V, and 4.05 V *vs.* Li^+^/Li. In subsequent cycles, a systematic peak shift of ∼30 mV and broadening are observed, particularly at high voltages indicating progressive polarization and structural instability above 4.1 V *vs.* Li^+^/Li. The single-layer LiNbO_3_ coated NCM811 (Fig. 5b) exhibits similar redox features with reduced overpotential and improved peak stability, although peak intensity fading persists. In contrast, the LiF|LiNbO_3_ dual-coated NCM811 (Fig. 5c) shows sharp, well defined and highly reversible redox peaks with the high voltage reduction peak at 4.11 V *vs.* Li^+^/Li (H3–H2) remaining stable over 10 cycles confirming structural robustness imparted by the dual-coating.

The Nyquist plots of the solid-state cells employing pristine NCM811, single-layer LiNbO_3_ coated NCM811 and dual-coated NCM811 with the LPSCl SE are shown in Fig. 5(d–f). The spectra were collected after charging the cells to 4.3 V *vs.* Li^+^/Li, where interfacial instability between high voltage cathodes and sulfide SE is typically most pronounced. In all cases, the impedance response is dominated by the SE/CAM interfacial resistance, indicating that charge–transfer processes across the cathode-electrolyte interface govern overall cell kinetics. The pristine NCM811 electrode exhibits a relatively large charge-transfer resistance of 350 Ω after the first charge, which increases dramatically to 730 Ω after 10 cycles. This pronounced impedance growth is attributed to direct chemical and electrochemical reactions between the highly oxidized NCM811 surface and the LPSCl SE, leading to the formation of resistive interphase products and impeded interfacial Li^+^ transport pathways.^7,9^

In contrast, the single-layer LiNbO_3_ coated NCM811 electrode shows a significantly reduced initial interfacial resistance of 230 Ω, increasing only slightly to 280 Ω after 10 cycles. LiNbO_3_ acts as a chemically stable interfacial buffer layer, suppressing direct contact between NCM811 and the LPSCl SE and thereby mitigating the interfacial decomposition reactions. Nevertheless, the gradual increase in resistance suggests that the protective layer may not fully cover the cathode surface, leaving localized regions vulnerable to electrolyte degradation, in line with the cross-section SEM/EDX reported in Fig. S11. Notably, the dual-coated NCM811 electrode exhibits the lowest interfacial resistance (155 Ω) and shows almost negligible increase after 10 cycles, indicating a highly stable SE/CAM interface. The superior stability is attributed to the dual layer architecture forming a more homogenous protective interface, effectively preventing parasitic reactions with the LPSCl SE and maintaining stable charge-transfer kinetics during cycling. Consequently, the dual-coated NCM811 demonstrates markedly improved interfacial stability, which is consistent with its enhanced electrochemical performance.

To address the interfacial beneficial effect of the LiF/LiNbO_3_ dual coating upon cycling, we have performed additional *ex situ* soft X-ray absorption spectroscopy (XAS) measurements at the Ni and Co L-edges using both total electron yield (TEY) and total fluorescence yield (TFY) detection modes at different states of charge during the first cycle. The corresponding results are presented in Fig. S12 and S13. The TEY mode probes the outermost cathode surface (≈10 nm depth) and is therefore highly sensitive to interfacial reactions occurring between NCM811 and the LPSCl SE, whereas TFY probes the near-surface/bulk region (hundreds of nanometers depth), enabling differentiation between surface degradation and bulk redox activity. The Ni L-edge TEY spectra (Fig. S12a and b) reveal significant differences between pristine and dual-coated NCM811. For the pristine uncoated NCM811 powder, the Ni^3+/4+^/Ni^2+^ ratio decreases from 0.83 to 0.70 after composite electrode fabrication and cell assembly, indicating the formation of additional Ni^2+^ species at the cathode surface as a result of spontaneous chemical reactions with LPSCl. Such interfacial reduction processes have been previously reported for sulfide-based ASSB batteries and are recognized as a major source of interfacial degradation and impedance growth. In contrast, the dual-coated LiF/LiNbO_3_ sample exhibits virtually no change in the Ni^3+/4+^/Ni^2+^ ratio (remaining ∼0.73 before and after cell assembly), demonstrating that the coating effectively mitigates the initial chemical reaction between NCM811 and LPSCl (Fig. S14 a).

Upon charging to 4.3 V *versus* Li^+^/Li, both materials exhibit substantial oxidation of Ni toward higher valence states in TEY (Fig. S12a and b) and TFY (Fig. S12c and d) modes, reaching comparable Ni^3+/4+^/Ni^2+^ ratios (Fig. S14a and b). This observation indicates that lithium extraction from the surface and bulk layered structure remains possible in both systems. However, significant differences emerge after discharge. The pristine NCM811 displays a higher residual Ni^3+/4+^/Ni^2+^ ratio in TEY, indicating incomplete lithiation and the persistence of electrochemically inactive surface regions. In contrast, the dual-coated NCM811 shows a more complete recovery of the Ni oxidation state, demonstrating improved reversibility of the surface redox processes. These results provide direct spectroscopic evidence that the dual coating mitigates the formation of electrochemically inactive surface species on NCM811 and preserves interfacial charge-transfer kinetics during cycling.

The change in the coverage of the LiNbO_3_ when changing the surface chemistry from Li_2_CO_3_ to LiF can be interpreted from general knowledge of sol–gel reactions. In sol–gel processes, the homogeneity and conformality of deposited layers are governed by interfacial electrostatic interactions between sol species and the particle surface, which are reflected in the zeta potential (the electrical potential at the slipping plane) and its relation to the isoelectric point (IEP). When the absolute zeta potential is sufficiently high, electrostatic repulsion suppresses particle aggregation and promotes uniform heterogeneous nucleation on individual particles. Conversely, when the surface charge approaches the IEP, reduced repulsion and dominant van der Waals attraction led to agglomeration, localized overgrowth, and patchy coatings.^32,33^ For high Ni layered oxides such as NCM811, surface structural reconstruction during air exposure produces residual lithium species (*e.g.*, LiOH and Li_2_CO_3_) that strongly modify surface acid base equilibria and shift the IEP.^34^ In particular, Li_2_CO_3_, as a basic and partially dissociative surface layer, alters the interfacial charge distribution, broadens the IEP distribution, and under typical acidic sol–gel conditions can reduce the magnitude of the zeta potential, thereby destabilizing the suspension and promoting heterogeneous, non-conformal deposition.^35^ In contrast, introducing a LiF interlayer fundamentally changes the surface charging behavior. LiF is chemically stable, highly insoluble and dominated by fluorine-terminated sites that are weakly polarizable and largely insensitive to protonation–deprotonation reactions. This homogenizes the IEP distribution and maintains a stable, predominantly neutral surface charge over a broad pH range, enhancing electrostatic stabilization and suppressing particle–particle agglomeration during coating. Consequently, the LiF inner layer acts as an electrostatic and chemical conditioning interface that facilitates controlled heterogeneous nucleation of the outer LiNbO_3_ sol–gel layer on individual NCM811 particles, leading to improved coating homogeneity, conformality, and thickness uniformity compared to Li_2_CO_3_ contaminated surfaces.

Beyond the observed improvements in coating morphology, the replacement of residual surface carbonates with a LiF interlayer may also alter the electronic landscape at the NCM811| LiNbO_3_ interface. Hypothetically, the highly ionic LiF could act as an electrostatic buffer that modifies the interfacial dipole. This would reduce the charge redistribution required for Fermi level alignment between the cathode and the coating, potentially mitigating the space-charge layer and “flattening” the electronic bands within the LiNbO_3_.^36^ However, confirming this electronic stabilization requires carefully decoupling it from the aforementioned morphological enhancements. Because the LiF pretreatment drastically improves the spatial homogeneity of the LiNbO_3_ overlayer, any observed improvements in interfacial kinetics or shifts in XPS binding energies could equally stem from the elimination of bare patches and uneven coating thicknesses. Future investigations—such as spatially resolved surface analyses or idealized thin-film model studies—will be necessary to definitively separate the intrinsic effects of the LiF interlayer on band bending from the macroscopic benefits of a uniform coating.

In this work we successfully fabricated a LiF/LiNbO_3_ dual protective coating on the surface of Ni-rich NCM811 cathodes through the consumption of residual lithium species during the fluorination process. GKD plots revealed that the dual coating is relatively more uniform and denser compared to the conventional single-layer LiNbO_3_ coating. In sulfide based solid-state cells, the dual-coated NCM811 cathode exhibited superior electrochemical performance including enhanced rate capability, improved long-term cyclability and significantly reduced interfacial charge-transfer resistance. These improvements originate from effective stabilization of the cathode interfaces, particularly at the CAM/SE interface, thereby mitigating detrimental chemical and electrochemical side reactions during cycling.

## Conflicts of interest

There are no conflicts to declare.

## Supplementary Material

TA-014-D6TA03220F-s001

## Data Availability

The data that support the findings of this study are openly available in Zenodo, reference number DOI: https://doi.org/10.5281/zenodo.19481888. Supplementary information (SI) is available. See DOI: https://doi.org/10.1039/d6ta03220f.
