# Supplementary material for: Overcoming cathode coating inhomogeneity: the role of LiF interlayer in enabling conformal LiNbO3 protection on LiNi0.8Co0.1Mn0.1O2 for all solid-state batteries
Source: J Mater Chem A Mater. 2026 Jul 24. Online ahead of print. doi: 10.1039/d6ta03220f (PMC13411624; doi:10.1039/d6ta03220f)
Supplement: TA-OLF-D6TA03220F-s001 [file TA-OLF-D6TA03220F-s001.pdf]

Supplementary Information (SI) for Journal of Materials Chemistry A.
This journal is © The Royal Society of Chemistry 2026

Supporting Information

Overcoming Cathode Coating Inhomogeneity: The Role of LiF interlayer in Enabling Conformal $LiNbO_3$ Protection on $LiNi_{0.8}Co_{0.1}Mn_{0.1}O_2$ for All Solid-State Batteries

Hari Vignesh Ramasamy,[a] Robin N. Wullich,[a] Barthélémy Lelotte,[a] Vallerie Siller,[a] Carlos F. Vaz,[b] Elisabeth Müller[c] and Mario El Kazzi *[a]

[a] PSI Center for Energy and Environmental Sciences, Paul Scherrer Institute, 5232 Villigen PSI, Switzerland

[b] PSI Center for Photon Science, Paul Scherrer Institute, 5232 Villigen PSI, Switzerland

[c] PSI Center for Life Sciences, Paul Scherrer Institute, 5232 Villigen PSI, Switzerland

Experimental Procedure

The polycrystalline $LiNi_{0.8}Co_{0.1}Mn_{0.1}O_2$ (NCM811) cathode was purchased from MSE Supplies with morphology composed of primary (~500 nm) and secondary particles (D50) of 10.0 $\pm$ 2.0 μm. Li_6PS_5Cl solid electrolyte was purchased from NEI corporation. The non hydrolytic sol-gel coating procedure using H_2O_2 and sonication has been adopted from our previous work.[1] Initially the NMC811 was mixed with ethanol with a concentration of 0.8g mL^{-1}. After complete dispersion, 1M lithium ethoxide solution (Sigma Aldrich) and a 0.5 M niobium ethoxide (Alfa Aesar, purity 99.999%) were added in a 1:1 molar ratio corresponding to 1, 2, 4 wt.% $LiNbO_3$ of NCM811. All the above process has been carried out inside the glovebox. The obtained suspension was sonicated for 30 min at room temperature at 80 kHz. Afterward, the solvent evaporated by heating at 100℃ overnight under dynamic vacuum. The resulting powder was grinded well using mortar and pestle and transferred to tube furnace. The powder was calcinated at 350℃ for 2 hours under oxygen flow of 25 L h^{-1}. The final samples are collected and stored in argon filled glovebox (O_2 and H_2O less than 1 ppm) for further use. The fluorination of the NCM811 surface was carried out as reported earlier in our previous work [2] except that the heating temperature is 350℃ for 1 hour. Surface fluorination of NCM811 was performed in a flow type reactor consisting of a silica equipped with a fritted silica disc for powder support. 3 g of NCM811 powder was loaded into the reactor in an Ar-filled glovebox and heated to 300 ℃ at a rate of 4℃/min under Ar flow (20 mL min^{-1}). Upon reaching the target temperature, a CHF_3/Ar gas mixture (1:1 volume ratio, total flow rate of 14 mL min^{-1}) was introduced and maintained for 1 hour. After fluorination, the reactor was evacuated and cooled to room temperature under dynamic vacuum. The fluorinated powder was subsequently transferred to an Ar-filled glovebox for further processing and characterization.

Cell fabrication

All the cathode powders were pre-dried at 120℃ in dynamic vacuum overnight. Custom made cells were dried in vacuum oven at 80°C overnight. These cells have an inner diameter of 7 mm. For the preparation of cathode composite, the NCM811 cathode, Li_6PS_5Cl and C65 were mixed using mortar and pestle in the wt% ratio of 70:29:1. Initially 23 mg Li_6PS_5Cl powder was pressed into pellet at 1.5 tons (382 MPa) for 1 min. 5 mg of cathode composite was added to one side of the SE pellet and pressed at 2 tons (510 MPa) for 1 min. For the counter electrode 7 mm disc of indium foil (0.1 mm thickness) is placed on other side of SE pellet followed by 3 mm disc of Lithium foil (0.2 mm thick). The atomic ratio of In:Li is 70:30. Copper foil and stainless steel is added as an anodic and cathodic current collector. Finally, the closed cell was cycled at a uniaxial stack pressure of 460 MPa, controlled using a torque wrench.

Electrochemical measurements

The assembled cells are subject to galvanostatic charge-discharge cycling between 2.7 V to 4.3 V vs Li^+/Li at varying current rates of C/10, C/4, C/2 and 1C (200 mAh g^{-1}). Cyclic voltammetry studies are carried at a scan rate of 0.05 mV s^{-1} within same voltage range. Electrochemical Impedance spectroscopy was measured after charge and discharge within frequency of 1 MHz to 10 mHz (10 mV). All these measurements are carried out at room temperature, and the assembled cells are maintained at open circuit potential (OCP) for 2 hours until cycling. These tests are performed using Biologic battery cycler.

Characterization techniques

Scanning Electron microscopy (SEM) Morphology of the samples are studied using Zeiss Ultra-55 microscope equipped with a field emission source and a 30-um aperture. The image was captured at an accelerated voltage of 5 kV and a working distance of 5mm. The images are captured in secondary electron mode. EDX mapping of different elements are performed using Oxford Ultim Extreme detector supported by Aztec software for data processing. The cross-section samples were prepared by an ion milling instrument Hitachi IM4000 using Ar-ion beam at room temperature, with an acceleration voltage of 4 kV and milling time of 5 hours for the pellets. All samples were transferred from the glovebox into the SEM chamber under vacuum using in-house built transfer chamber.

A detailed description of Gaussian Kernel density (GKD) mapping of EDX data were provided in our previous work.[1]

X-ray photoelectron spectroscopy (XPS) measurements. For this we used VG ESCALAB 220iXL spectrometer (Thermo Fischer Scientific) equipped with a monochromated focused Al K-alpha source (1486.6 eV) with a beam size of 500 µm in diameter. Survey spectra were recorded using 70 eV pass energy, step energy of 0.05 eV and a dwell time of 20 ms, while high resolution core level spectra were recorded with a pass energy of 20 eV , energy step of 0.5 eV and a dwell time of 50 ms. XPS samples are prepared by mixing NMC811 cathode with 10 % C65 carbon and mounted as powder on a stainless steel sample holder using conductive copper tape to omit charging effects. All data obtained were calibrated with respect to C1s core level spectra as 248.8 eV. Fitting was carried out using CasaXPS software (Copyright Casa Software Ltd). The deconvolution of the components was carried out using a Shirley-type background subtraction has been used along with a sum of Gaussian (70) and Lorentzian peak (30) shapes in the Marquardt fit method under application of a relative sensitivity factor of 1. Residual standard deviation is close to unity for Nb5d and O1s peaks. For Nb3d, the spin-orbit split (ΔE) and branching ratio (β) of the $Nb3d_{5/2+3/2}$ $\Delta E_{Nb3d} = 2.72$ eV, and $\beta_{Nb3d} = 1.48$. The list of parameters used in fitting was provided in Table S1 and Table S2.

Transmission Electron Microscopy (TEM) The fluorinated NCM811 samples was loaded to a focused ion beam tool (FIB; Zeiss NVision40) tool for the preparation of a 50 – 200 nm thick TEM lamella. The particle was coated by carbon and platinum layers by electron-assisted deposition followed by Ga-assisted deposition to maintain the sample integrity during Ga-ion milling. The final polishing step was done with a Ga-ion beam of 100 pA and 3keV acceleration voltage. For this analysis, a probe corrected JEOL JEM-ARM200F instrument equipped with a cold-field emission gun (cold FEG) and a JEOL energy-dispersive X-ray (EDX) detector was used. Additionally, scanning transmission electron microscopy (STEM)-EDX mapping and point and area spectral acquisition were performed to qualitatively identify the elemental composition of the particles and surface coating, specifically targeting, Ni, Co, Mn, Nb and F.

Ex situ X-ray absorption spectroscopy (XAS): X-ray absorption spectroscopy (XAS) measurements were performed at the SIM beamline of the Swiss Light Source (SLS), Paul Scherrer Institute. Following the ASSB cell disassembly in an argon-filled glovebox, the working electrodes were transferred to the beamline using an air-tight transfer chamber to prevent exposure to ambient atmosphere. The SIM beamline operates within a photon energy range of 0.1 to 2 keV, enabling access to the Ni, Co, Mn, S, and P L2,3-edges, O, and C K-edges. During data acquisition, the residual pressure

inside the spectroscopy chamber was maintained under ultra-high vacuum (UHV) at approximately 10^{-9} mbar.

XAS spectra were primarily acquired in total fluorescence yield (TFY) detection mode using a silicon diode (AXUV100AL, Opto Diode) positioned at an angle of 45° relative to the sample surface. TFY is sensitive to near surface region, probing depth approximately ~100s nm depending on the absorption edge and material composition.

The total electron yield (TEY) detection mode spectra were recorded by collecting the secondary electrons emitted by the sample as a function of photon energy by measuring the sample electron current via a picoammeter (Keithley 6517B). As a rule of thumb in TEY mode the probed depth is (~10 nm). Combining TEY and TFY detection mode allows a non-destructive depth profiling from the surface to the bulk of the probed particles.

XAS data processing was carried out by normalizing all spectra to the incident X-ray flux (I_0), measured using either a gold mesh or the beamline focusing mirror. Following normalization, all spectra obtained were processed by subtracting a linear pre-edge background and subsequently normalized to the intensity of a characteristic absorption feature to facilitate quantitative comparison between different

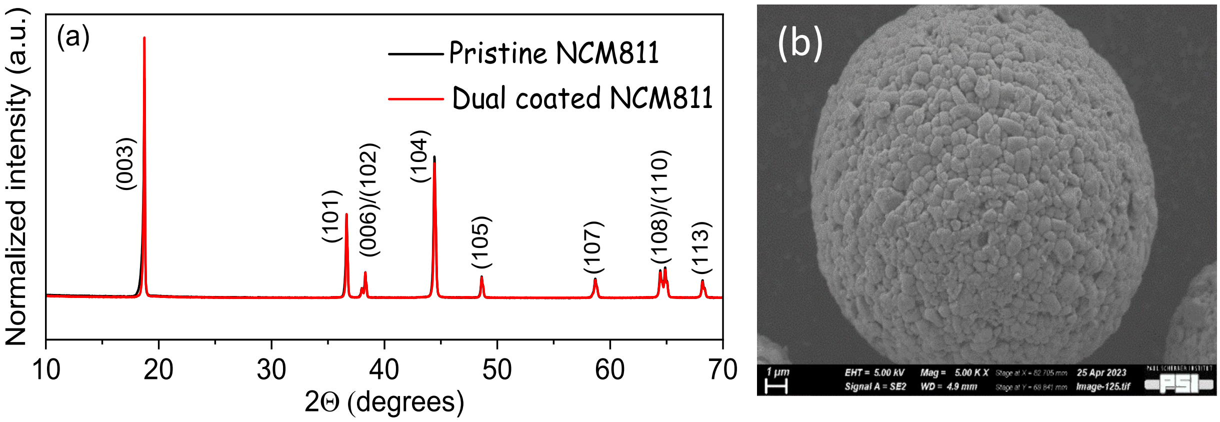

electrochemical states.

Fig. S1 (a) XRD peaks of pristine and LiF|$LiNbO_3$ dual-coated NCM811 cathode, (b) SEM image of the pristine NCM811 cathode.

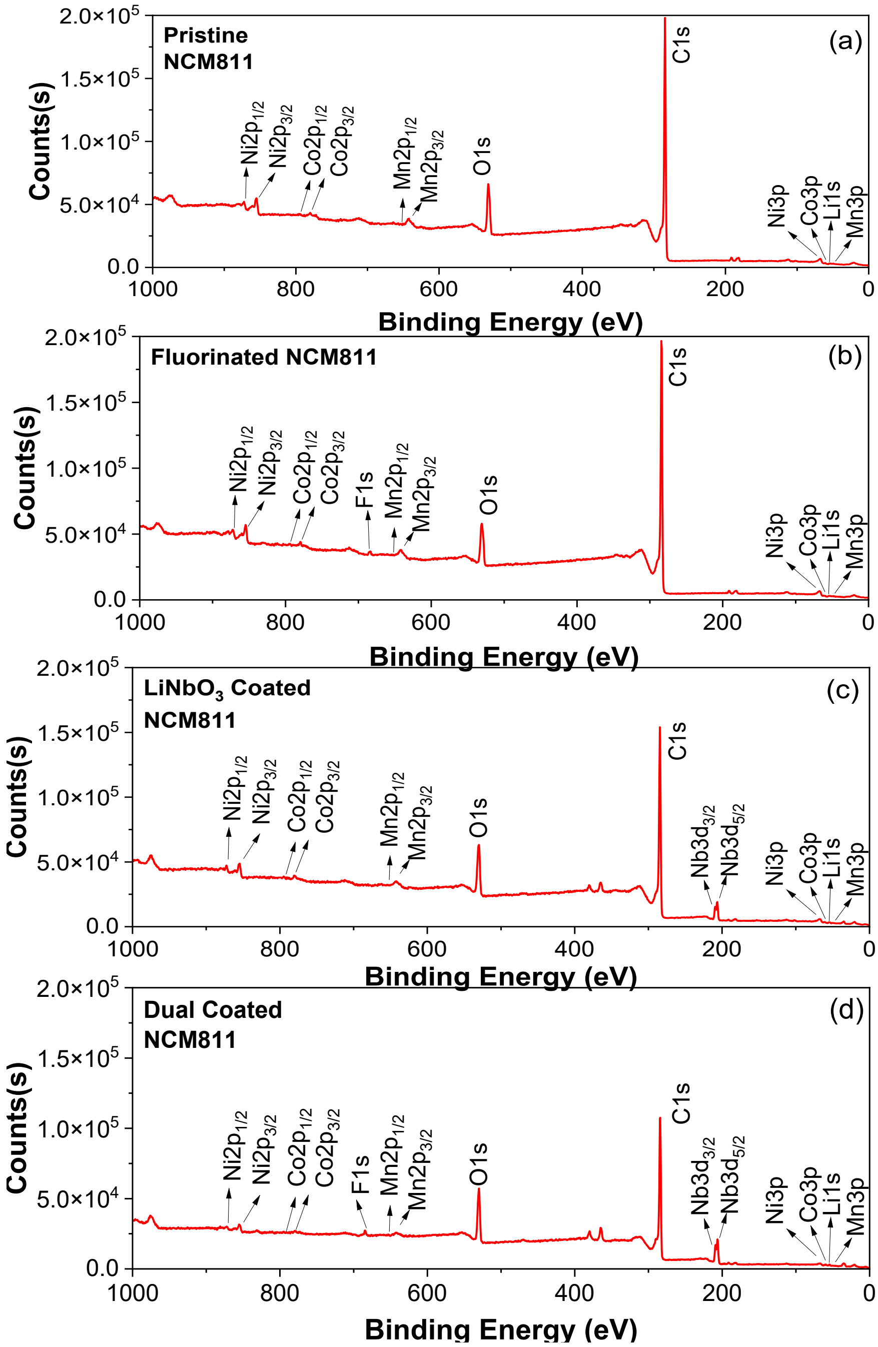

Fig. S2. XPS survey spectrum of (a) pristine (b) fluorinated (c) single-layer $LiNbO_3$ coated (d) LiF|$LiNbO_3$ dual-coated NCM811.

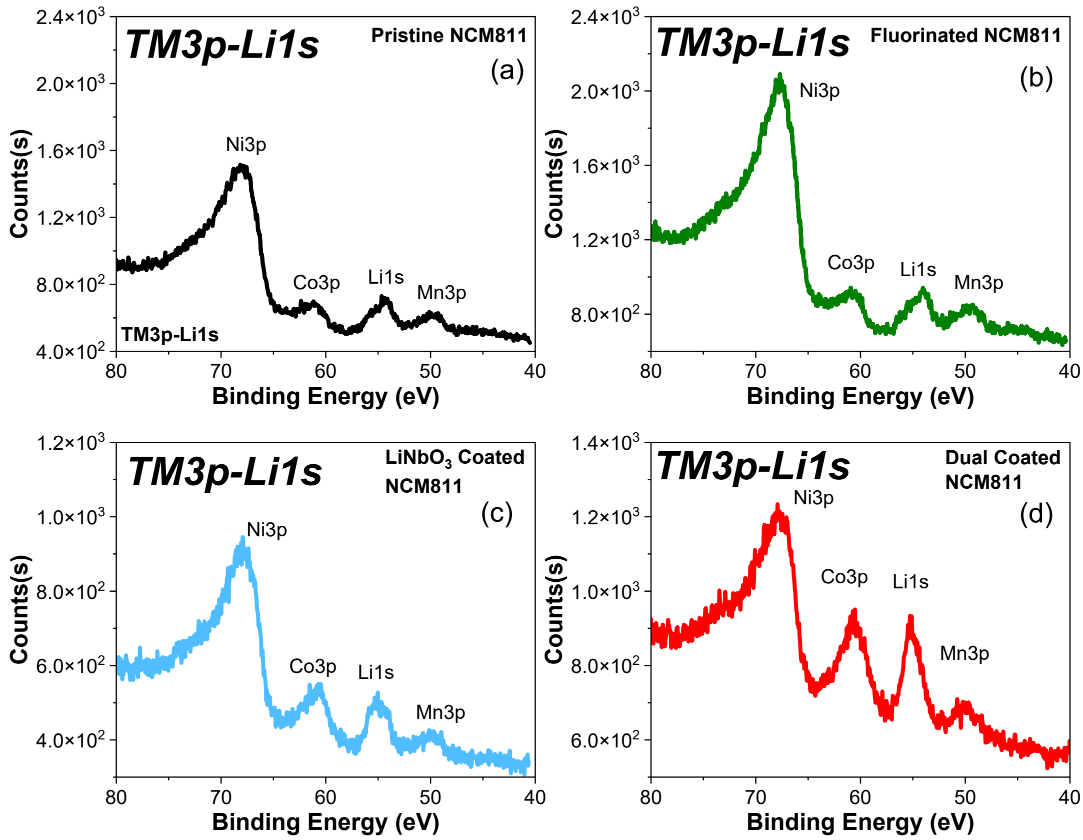

Fig. S3 Ni3p, Co3p, Mn3p and Li1s XPS core level spectra measured on (a) pristine, (b) fluorinated, (c) single-layer $LiNbO_3$ coated and (d) LiF|$LiNbO_3$ dual-coated NMC811.

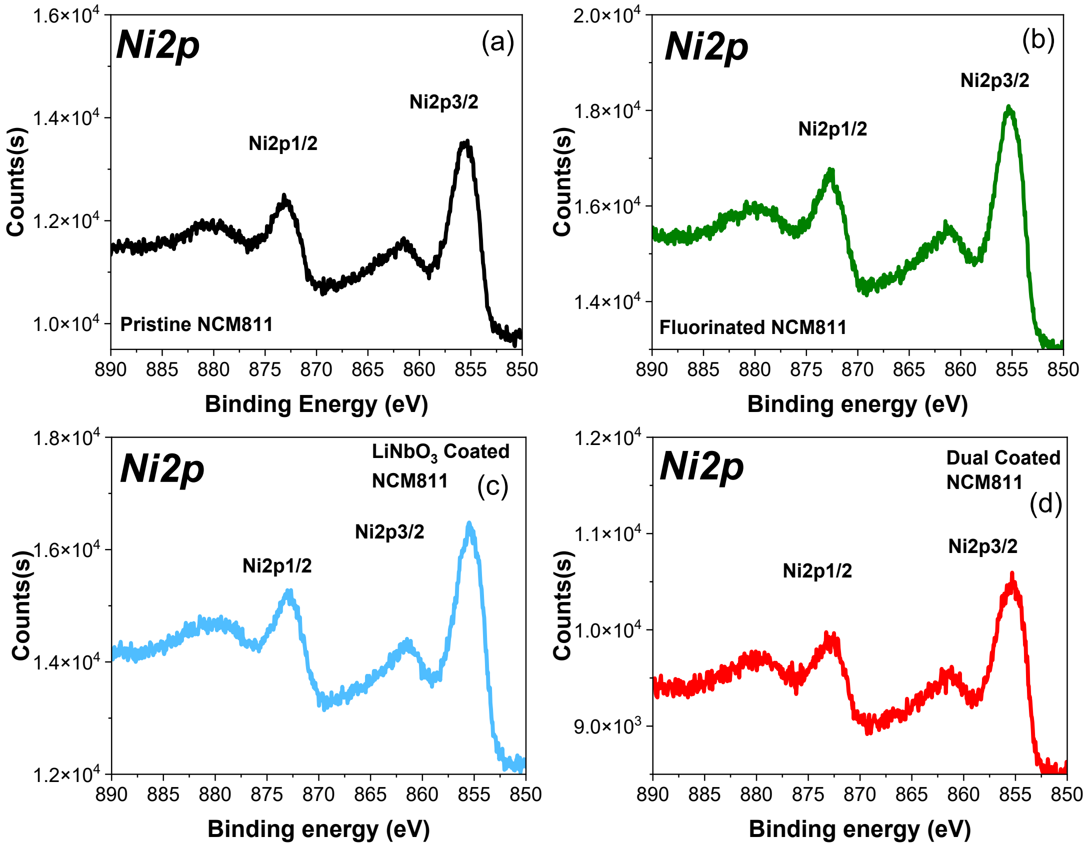

Fig. S4 Ni2p XPS core level spectra measured on (a) pristine, (b) fluorinated, (c) single-layer $LiNbO_3$ coated and (d) LiF|$LiNbO_3$ dual-coated NCM811.

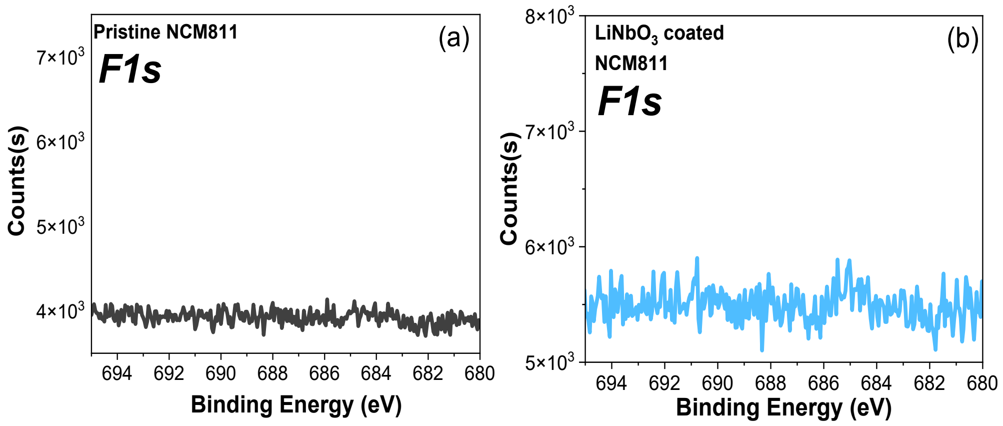

Fig. S5 F1s XPS core level spectrum measured on (a) pristine and (b) single-layer $LiNbO_3$ coated NCM811.

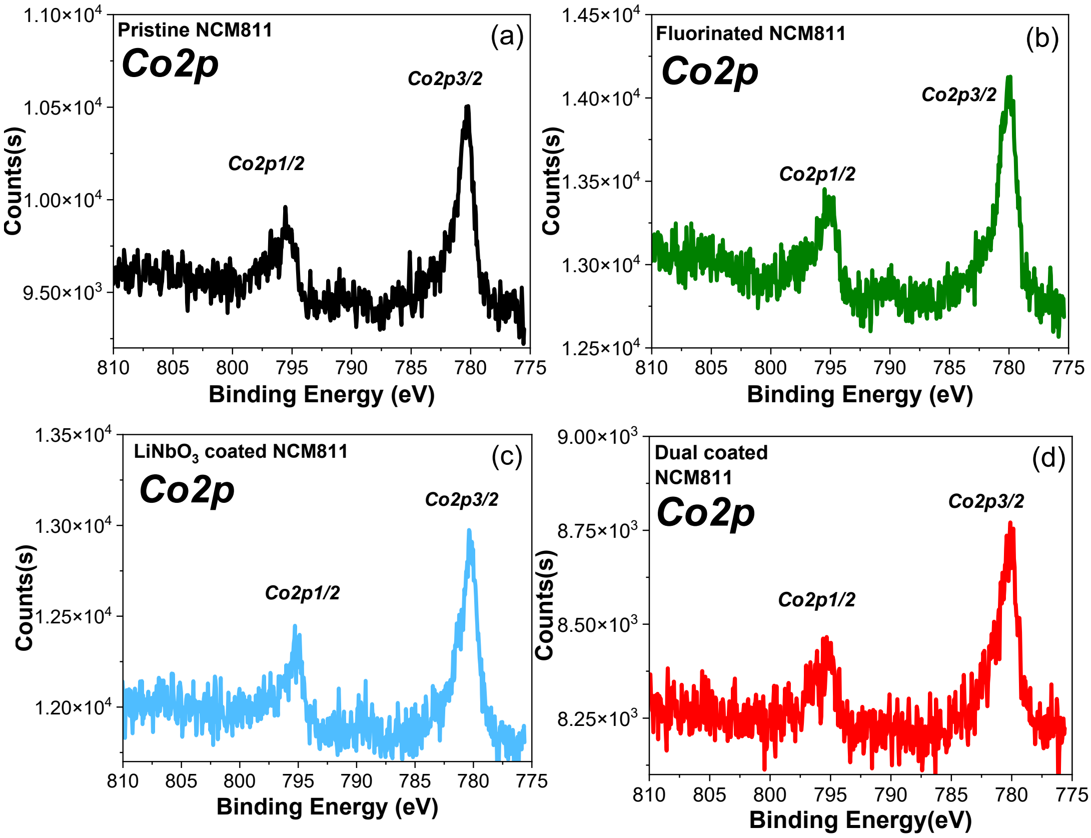

Fig. S6 Co2p XPS core level spectrum measured on (a) pristine, (b) fluorinated, (c) single-layer $LiNbO_3$ coated and (d) LiF|$LiNbO_3$ dual-coated NCM811.

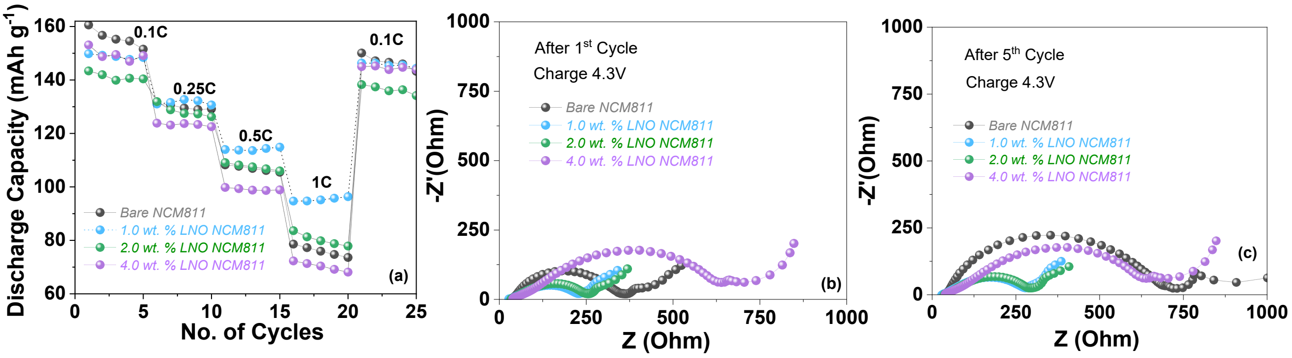

Fig. S7 (a) Rate capability with 1, 2 and 4 wt.% of single-layer $LiNbO_3$ coating on NCM811 cathode (b) Nyquist plot of the measured impedance spectroscopy after 1st charge to 4.3 V vs Li^+/Li, (c) after 5th Charge.

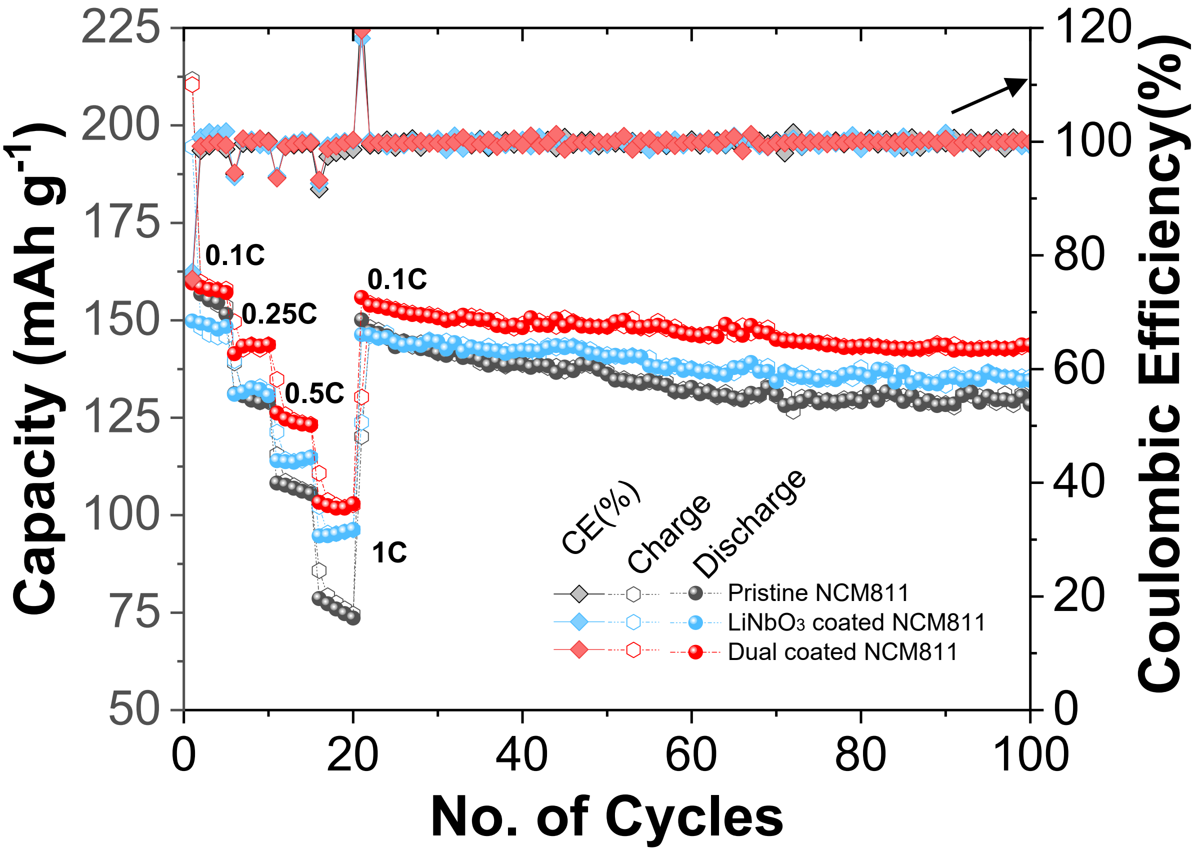

Fig. S8 Rate capability and long-term cycling performance measured at varying current density (from 0.1C to 1C) between 2.1 V to 3.7 V vs $InLi_x$ (2.7 V to 4.3 V vs Li^+/Li) for pristine NCM811, single-layer $LiNbO_3$ coated NCM811 and LiF|$LiNbO_3$ dual-coated NCM811.

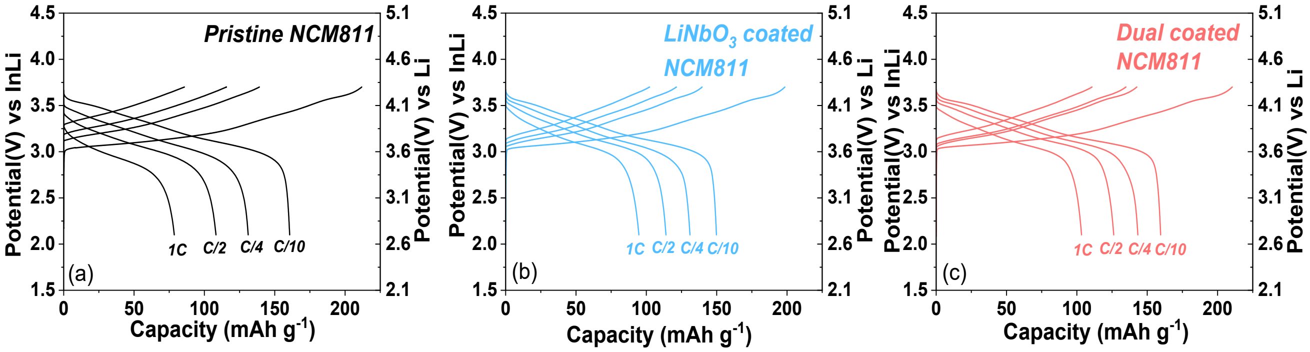

Fig. S9 Galvanostatic charge and discharge curves measured at different current rates between 2.1 V to 3.7 V vs $InLi_x$ (2.7 V to 4.3 V vs Li^+/Li) for (a) pristine (b) single-layer $LiNbO_3$ coated and (c) LiF|$LiNbO_3$ dual-coated NCM811.

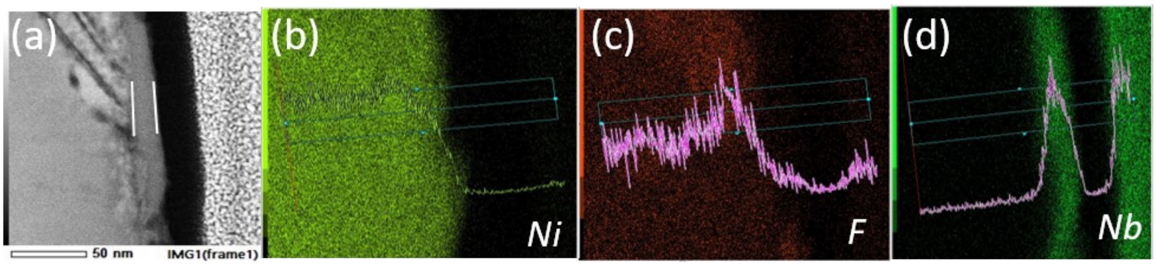

Figure S10. (a) Dark field (DF) STEM image taken on dual LiF|$LiNbO_3$ coated NCM811 along with EDX mapping overlaid with linescan of the elements (b) Ni Kα, (c) F Kα, and (d) Nb Lα.

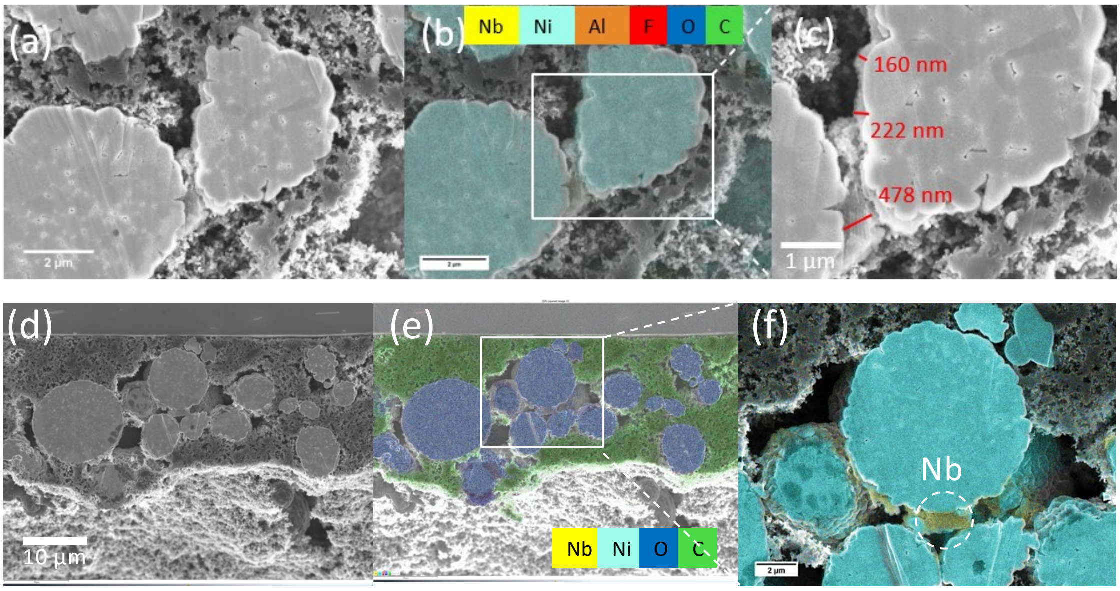

Figure S11. Cross-sectional Ar-ion-milled performed on working electrode composed of 80 wt.% single-layer $LiNbO_3$-coated NCM811, 10 wt.% C65 conductive carbon, and 10 wt.% PVDF binder. (a, c, d) SEM cross-sectional images and the corresponding (b, e and f) EDX elemental maps of Nb Lα, Ni Lα, O Kα, and C Kα. The Nb distribution reveals a heterogeneous $LiNbO_3$ coating morphology with localized Nb-rich agglomerates and significant thickness variations across the NCM811 particle surface, indicating non-uniform coating coverage in the absence of the LiF interlayer.

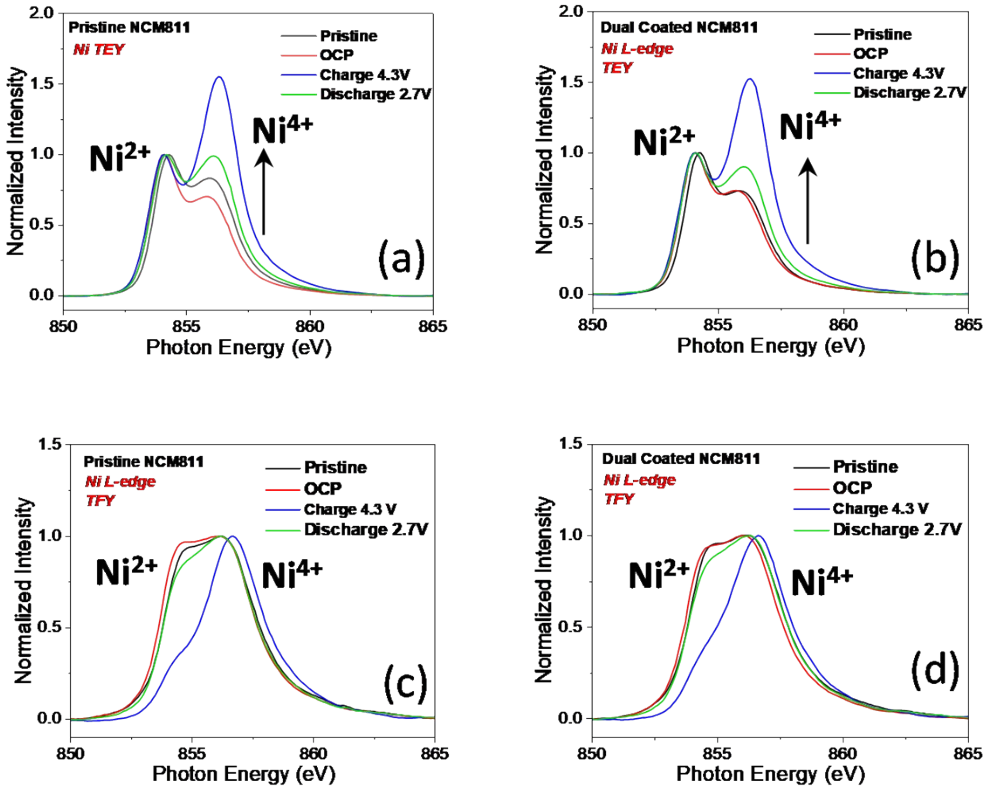

Fig S12. Ex situ Ni L-edge XAS spectra acquired in (a,b) total electron yield (TEY) and (c,d) total fluorescence yield (TFY) detection modes for pristine NCM811 and dual-coated NCM811 powders, as well as for composite working electrodes composed of 70 wt.% pristine or dual-coated NCM811, 29 wt.% LPSCl, and 1 wt.% C65 conductive carbon. Spectra were collected at OCP, after the first charge to 4.3 V vs. Li^+/Li, and after the first discharge to 2.7 V vs. Li^+/Li.

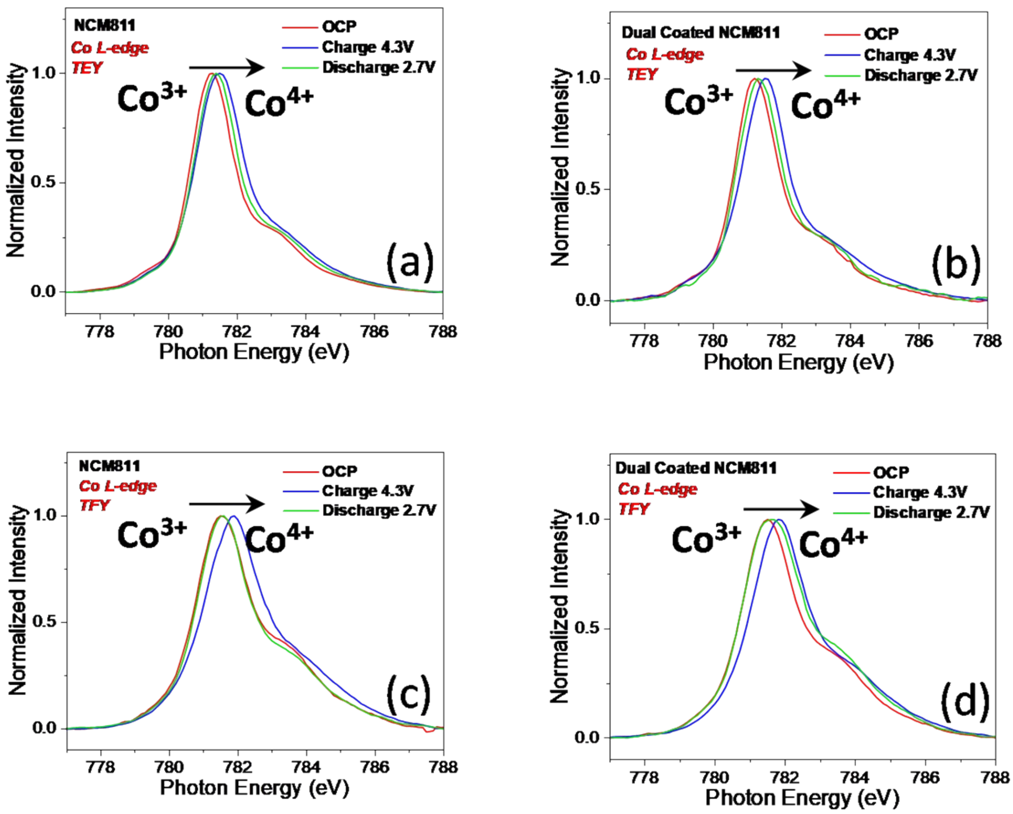

Fig S13. Ex situ Co L-edge XAS spectra acquired in (a,b) total electron yield (TEY) and (c,d) total fluorescence yield (TFY) detection modes for composite working electrodes composed of 70 wt.% pristine or dual-coated NCM811, 29 wt.% LPSCl, and 1 wt.% C65 conductive carbon. Spectra were collected at OCP, after the first charge to 4.3 V vs. Li^+/Li, and after the first discharge to 2.7 V vs. Li^+/Li.

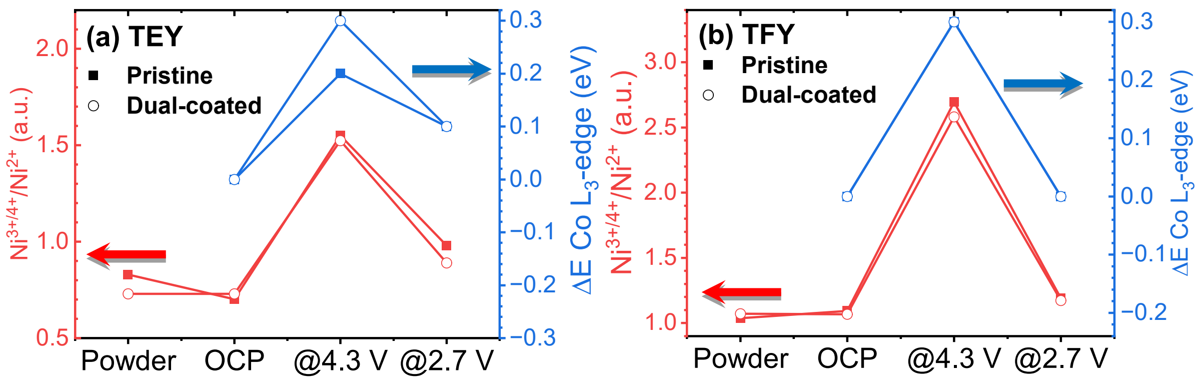

Fig S14. Evolution of the Ni^{4+}/Ni^{2+} intensity ratio and Co L_3-edge energy shift ($\Delta E_{Co\ L3}$) derived from the Ni and Co L_3-edge XAS spectra, respectively. Data were acquired in (a) total electron yield (TEY) and (b) total fluorescence yield (TFY) detection modes for pristine NCM811 powders and composite working electrodes composed of 70 wt.% pristine or dual-coated NCM811, 29 wt.% LPSCl, and 1 wt.% C65 conductive carbon. Measurements were performed at OCP, after the first charge to 4.3 V vs. Li^+/Li, and after the first discharge to 2.7 V vs. Li^+/Li. The Ni^{4+}/Ni^{2+} ratio and $\Delta E_{Co\ L3}$ provide quantitative indicators of the transition-metal oxidation state evolution during electrochemical cycling and enable comparison of the surface-sensitive (TEY) and near-surface/bulk-sensitive (TFY) responses of pristine and coated cathode materials.

Deconvolution Parameters (O1s)		Samples			
		Pristine NCM811	Fluorinated NCM811	$LiNbO_3$ coated NCM811	Dual coated NCM811
Binding Energy (eV)	O-lattice	529.22	529.02	529.22	529.22
	CO^{2-}_3	531.39	531.44	531.48	531.48
	C=O	532.22	532.27	532.21	532.21
	C-O	533.76	533.69	530.2	-
	O-depleted	529.79	530.00	-	
	$LiNbO_3$	-	-	530.20	530.12
FWHM	O-lattice	1.072	1.10	1.0	1.0
	CO^{2-}_3	1.93	1.89	1.95	1.9
	C=O	1.946	1.95	1.9	1.95
	C-O	1.97	1.97	1.974	
	O-depleted	1.6	1.6	-	
	$LiNbO_3$	-	-	1.472	1.3116

Table S1 List of deconvolution parameters used for CasaXPS fitting of O1s peaks

Deconvolution Parameters (Nb5d)		Samples			
		Pristine NCM811	Fluorinated NCM811	$LiNbO_3$ coated NCM811	Dual coated NCM811
Binding Energy (eV)	$Nb5d_{3/2}$	-	-	210.15	209.62
	$Nb5d_{5/2}$	-	-	207.40	206.90
FWHM	$Nb5d_{3/2}$	-	-	1.322	1.377
	$Nb5d_{5/2}$	-	-	1.285	1.299

Table S2 List of deconvolution parameters used for CasaXPS fitting of Nb5d peaks

References

1. R. N. Wullich, B. Lelotte, V. Pelé, C. Jordy, L. Gubler and M. El Kazzi, *Electrochim. Acta*, 2025, **536**, 146750
2. A. Štefančič, C. A. F. Vaz, D. Baster, E. Müller and M. El Kazzi, *ChemSusChem*, 2025, **18**, 1–12.
